# Anthropogenic Infection of Domestic Cats With SARS-CoV-2 Alpha Variant B.1.1.7 Lineage in Buenos Aires

**DOI:** 10.3389/fvets.2022.790058

**Published:** 2022-03-01

**Authors:** Andrea Pecora, Dario Amilcar Malacari, Marina Valeria Mozgovoj, María de los Ángeles Díaz, Andrea Verónica Peralta, Marco Cacciabue, Andrea Fabiana Puebla, Cristian Carusso, Silvia Leonor Mundo, María Mora Gonzalez Lopez Ledesma, Andrea Vanesa Gamarnik, Osvaldo Rinaldi, Osvaldo Vidal, Javier Mas, María José Dus Santos

**Affiliations:** ^1^Centro de Investigaciones en Ciencias Veterinarias y Agronómicas, Instituto de Virología e Innovaciones Tecnológicas, Instituto Nacional de Tecnología Agropecuaria (INTA)-Consejo Nacional de Investigaciones Científicas y Técnicas (CONICET), Buenos Aires, Argentina; ^2^Diagnogen S.A., Buenos Aires, Argentina; ^3^Instituto de Biotecnología, Universidad Nacional de Hurlingham, Buenos Aires, Argentina; ^4^Centro de Agroindustria, Instituto de Ciencia y Tecnología de Sistemas Alimentarios Sustentables, Instituto Nacional de Tecnología Agropecuaria (INTA)-Consejo Nacional de Investigaciones Científicas y Técnicas (CONICET), Buenos Aires, Argentina; ^5^Secretaria de Salud de la Municipalidad de la Matanza, Ministerio de Salud, Buenos Aires, Argentina; ^6^Centro de Investigaciones en Ciencias Veterinarias y Agronómicas, Instituto de Agrobiotecnología y Biología Molecular, Instituto Nacional de Tecnología Agropecuaria (INTA)-Consejo Nacional de Investigaciones Científicas y Técnicas (CONICET), Buenos Aires, Argentina; ^7^Facultad de Ciencias Veterinarias, Universidad de Buenos Aires, Buenos Aires, Argentina; ^8^Instituto de Investigaciones Bioquímicas de Buenos Aires (IIBBA, CONICET-Fundación Instituto Leloir), Buenos Aires, Argentina; ^9^Veterinaria Rinaldi Vidal, Buenos Aires, Argentina; ^10^Diagnotest SRL, Buenos Aires, Argentina

**Keywords:** SARS-CoV-2, COVID-19, reverse zoonosis, cats, One Health

## Abstract

SARS-CoV-2 reverse zoonosis, particularly to domestic animals, and the potential role of infected animals in perpetuating the spread of the virus is an issue of increasing concern. In this case report, we identified the natural infection of two cats by SARS-CoV-2, in Argentina, whose owner had been previously infected by SARS-CoV-2. Viral genetic material was detected in feline oropharyngeal (OP) and rectal (R) swab by RT-qPCR, and sequence analysis revealed that the virus infecting the owner and one cat were genetically similar. The alpha variant (B.1.1.7 lineage) was identified with a unique additional mutation, strongly suggesting human-to-cat route of transmission. This study reinforces the One Health concept and the importance of integrating human, animal, and environmental perspectives to promptly address relevant health issues.

## Introduction

SARS-CoV-2 is an emergent coronavirus and the cause of coronavirus disease 2019 (COVID-19). The pandemic caused by SARS-CoV-2 has reached almost all countries in the world with an extraordinary person-to-person transmission rate (1). Moreover, it represents the most important global pandemic of an emerging zoonotic disease in this century.

Coronaviruses are members of the subfamily *Coronavirinae*, family *Coronaviridae*, order Nidovirales, and are involved in human and vertebrate diseases. Based on its phylogenetic relationships and genomic structures, SARS-CoV-2 belongs to the genera Betacoronavirus (together with SARS-CoV and MERS-CoV) (2, 3). Betacoronavirus has a broad host range including different vertebrate groups (human, bovine, feline, canine, equine, and camelids), cause important respiratory, enteric, and systemic infectious diseases (4), and they can eventually cross the species barriers generating spillover events (5).

It is reported that some wild and companion animals are susceptible to infection by SARS-CoV-2. For this reason, although human-to-human transmission is the main way of virus spreading, there is an increasing concern regarding reverse zoonosis, particularly to domestic animals, and the potential role that infected animals could play in perpetuating the spread of the disease (6).

The first report of reverse zoonosis, was a dog from Hong Kong (7). After that, other cases of domestic cats and dogs, wild felids (i.e., lion and tigers at the Bronx Zoo, USA), and minks becoming infected following exposure to infected owners were reported (6, 8, 9).

Since the beginning of the pandemic, domestic cats infected with SARS-CoV-2 have been identified in many countries including China (10), Belgium (11), Italy (12), UK (13), France (14), Spain (15), Brazil (16), USA (8), and Argentina (9). In addition, animal susceptibility experiments demonstrated that dogs, cats, ferrets, pigs, chickens, and ducks can be experimentally infected with SARS-CoV-2 (17) and that cats and ferrets are the most susceptible species.

Regarding infection of SARS-CoV-2 in animals, most of the studies indicate that the clinical signs were respiratory disease and/or conjunctivitis (8, 11, 14, 18), but in some other reports, cats and dogs were asymptomatic. Results from experimental infections indicated that cats can shed the virus by the oral and nasal route during a prolonged period of time, but they mostly remained asymptomatic; in addition, infected cats developed a robust neutralizing antibody response and were capable of transmitting the disease to other cats (8, 19–21). However, reported cases of felines infected with SARS-CoV-2 were related to close contact with humans diagnosed with COVID-19, and disease occurrence in cats is actually considered sporadic.

In this case report, we identified the natural infection of two cats by SARS-CoV-2, in Argentina, whose owner had been previously infected by SARS-CoV-2. The SARS-CoV-2 genetic material was detected in feline oropharyngeal (OP) and rectal (R) swabs by RT-qPCR and sequence analysis revealed that the virus infecting the owner and one cat were genetically related. The alpha variant (B.1.1.7 lineage) was identified with a unique additional mutation, strongly suggesting the human-to-cat route of transmission.

## Methods

### Human Component

#### Clinical Case

On April 10, a 39 year-old man noticed general discomfort. Other symptoms included cough, fever, headache, and anosmia; all clinical signs were consistent with the coronavirus disease (COVID-19). Two days after the onset of symptoms, the human patient was sampled for SARS-CoV-2 diagnosis. He did not present any comorbidities and had not been vaccinated for SARS-CoV-2.

He lived with his wife (41 years old) and daughter (18 years old), who started after 2 days with fever, general discomfort, odynophagia, and headache. They were regarded as COVID-positive cases according to epidemiological clinical criteria, but they were not tested by RT-PCR. The family shared a two-room apartment (located in a district in the west of the Metropolitan Area of Buenos Aires) with four feline pets.

#### Detection of SARS-CoV-2

The nasopharyngeal (NP) swab from the man was collected at the San Juan de Dios Hospital. For routine diagnosis, viral RNA was extracted from 200 μl of sample using High Pure Viral Nucleic Acid Kit (Roche) and according to the instructions of the manufacturer. A commercial one-step reverse transcription real-time polymerase chain reaction (Tib-Molbiol, Roche) was performed to confirm the presence of SARS-CoV-2 by amplification of RdRp and E genes from extracted RNA, according to the instructions of the manufacturer. RT-qPCR assays were carried out using LightCycler Multiplex RNA Virus Master Mix (Roche) on the LightCycler 2.0 (Roche) instrument. RNA was stored at −80°C.

### Animal Component

The house was inhabited by four adopted stray cats that lived indoors with the humans:

Cat N°1 was a 2-year-old neutered male, 3.6 kg, European domestic shorthair; Cat N°2 was a 5-year-old female, 3.2 kg, European domestic shorthair; Cat N°3 was a 4-year-old female, 3.2 kg, European domestic shorthair; and Cat N°4 was a 13-year-old neutered male, 4.7 kg, European domestic shorthair.

#### Clinical Examination

Physical examination involved observing the appearance of the cats, auscultation, palpation, rectal temperature, dehydration degree, and mucosal examination. Anamnesis and complementary studies were performed, including thoracic radiographs for Cat N°3.

For specimen collection, the cats were sedated by an intramuscular injection of a combination of ketamine hydrochloride (10 mg/kg) and acepromazine maleate (0.1 mg/kg). A total of 3–5 ml of blood was collected by cephalic vein venipuncture to obtain serum for serologic complementary studies.

The OP and R swabs were collected with DACRON swabs and placed in a single 15-ml sterile tube containing 3 ml of Viral Transport Medium (VTM). After sampling, all VTM tubes were transferred to the laboratory on the same day where the VTM was stored at −70°C.

All samples were obtained and conditioned according to the guide for the detection of SARS-CoV-2 in animals (https://www.argentina.gob.ar/sites/default/files/covid-19-guia-para-deteccion-sars-cov-2-en-animales.pdf). The owner gave a written consent to allow the sampling of their pet cats.

#### SARS-CoV-2 Serology in Cats

SARS-CoV-2 IgG antibodies were tested by an indirect ELISA using the Argentina kit COVID AR IgG ELISA test (Laboratorio Lemos S.R.L.) adapted for domestic feline samples (19). In the assay, anti-human IgG was replaced by a peroxidase-conjugated goat anti-cat IgG [Goat anti-Feline IgG (H+L) HRP, Invitrogen A18757], used at a 1:20,000 dilution. All steps were conducted following the instructions of the manufacturer. The optical density (OD) was measured at 450/630 nm.

Neutralization assays were carried out with SARS-CoV-2 pseudotyped particles (CoV2pp-GFP), generated in Sean Whelan laboratory (20). CoV2pp-GFP carries vesicular stomatitis virus as viral backbone, bearing the E gene in place of its G glycoprotein (VSV-eGFPSARS-CoV-2), and expresses full-length wild-type or mutant spike variant on its envelope. Vero cells were used for these assays. Cells were maintained with DMEM high glucose with 10% FBS and were seeded in a 96-well plate on the day before infection. Patient sera were heat inactivated at 56°C for 30 min and serially diluted in DMEM high-glucose medium. Serum neutralizations were performed by first diluting the inactivated sample two-fold and continuing with a two-fold serial dilution. A pretitrated amount of pseudotyped particles was incubated with a two-fold serial dilution of patient sera for 1 h at 37°C prior to infection. Subsequently, cells were fixed in 4% formaldehyde containing 2 mg/ml of DAPI nuclear stain (Invitrogen) for 1 h at room temperature, and fixative was replaced with PBS. Images were acquired with the InCell 2000 Analyzer (GE Healthcare) automated microscope in both the DAPI and FITC channels to visualize nuclei and infected cells (i.e., eGFP-positive cells), respectively, ( × 4 objective, four fields per well, covering the entire well). Images were analyzed using the Multi Target Analysis Module of the InCell Analyzer 2000 Workstation Software (GE Healthcare). GFP-positive cells were identified in the FITC channel using the top-hat segmentation method and subsequently counted within the InCell Workstation software (19). As controls, positive and negative cat sera were included in the assay.

Serum samples from Cats N°2, N°3, and N°4 were subjected to a commercial immunochromatography test to detect antibodies against feline coronavirus, following the instructions of the manufacturer (Speed Assist F-Corona, Virbac).

#### Detection of SARS-CoV-2 RNA in Cats

Viral RNA was extracted directly from OP and R swab samples using High Pure Viral RNA kit (Roche) according to the recommendations of the manufacturer. All samples were tested by a commercial *in vitro* diagnostic test (RT-qPCR) designed to amplify the SARS-CoV-2 ORF region (GENESIG), according to the instructions of the manufacturer. The PCR detects SARS-CoV-2 but not other coronaviruses.

### SARS-CoV-2 Viral Load Quantification

Levels of SARS-CoV-2 viral load (VL) were quantified using the set of primers and probe for SARS-CoV-2 E gene described by Corman et al. (5) (E_Sarbeco_F; E_Sarbeco_R; E_Sarbeco_P1). Each reaction contained 5 μl of RNA, 12.5 μl of qScript® XLT One-Step RT-qPCR ToughMix (Quantabio), 400 nM of each primer, and 200 nM of probe.

The E gene fragment amplified using E_Sarbeco_F and E_Sarbeco_R primers was inserted in a pGEM-T easy vector (Promega). The standard curve was performed with 10-fold dilutions of that plasmid (10^6^-10^1^ genomic copies/μl). The assay was run in triplicate for each sample and each point of the standard curve and showed an efficiency of 100.2%. Viral load (VL) was expressed as genomic copies/μl of sample. RNA from positive and negative human samples were included as controls of the procedure.

### Sequencing and Phylogenetic Analysis

Complete SARS-CoV2 genome sequences were obtained using the Quick protocol (Quick 2020) and Oxford Nanopore platform. Briefly, cDNA was synthesized with SuperScript III Reverse Transcriptase (ThermoFisher). Then the multiplex developed by Artic Network was performed. Amplicons A and B were visualized on 1.5% agarose gel, purified using AMPure beads, and DNA was quantified by Qubit 2.0 Fluorometer. The libraries were built following the Oxford Nanopore specifications.

ARTIC amplicons for Nanopore were prepared using the ONT Native Barcoding Expansion kit (EXP-NBD104). Up to 24 samples were multiplexed on a flow cell and sequenced on a MinION. The ONT MinKNOW software was used to collect raw sequencing data. The *RAMPART* (v1.2.0) software package was used to monitor sequencing performance in real-time (22). Next, the bioinformatics protocol established by the ARTIC network for viral surveillance was followed (https://artic.network/ncov-2019/ncov2019-bioinformatics-sop.html). Briefly, the resulting reads were basecalled using Guppy (v5.0.11). The *guppy_barcoder* tool was used for demultiplexing and adapter trimming. Low-quality reads (mean quality score <7), reads shorter than 400 bp and reads longer than 600, were filtered using the *artic guppyplex* tool. High-quality reads were then aligned to the Wuhan-Hu-1 reference genome (GenBank accession number MN908947.3) using minimap2 (v2.17) (23), and the alignment files were processed with *SAMtools* (24). The *align_trim* tool was used to trim primer sequences from the read alignments and normalize sequencing depth at a maximum of 400-fold coverage. Consensus-level variant candidates were identified using *Medaka* (v1.0.3), evaluated by *LongShot* (v0.4.1) (25), and filtered with the *artic_vcf_filter* tool.

The phylogenetic analysis of SARS-Cov2 whole-genome sequences of alpha (lineage B1.1.7) was carried out with Argentine Alpha sequences deposited in the GISAID EpiCoVdatabase. Briefly, the consensus-level sequences were aligned to the reference genome using *Mafft* (v7.487) (26). A maximum likelihood phylogenetic tree was constructed using *IQ-TREE*(v1.6.12) (27). The consensus tree was visualized and exported with *FigTree* (v1.4.4) (http://tree.bio.ed.ac.uk/software/figtree/).

## Results

### SARS-CoV-2 Clinical Presentation and Molecular Detection

**Figure 1:**
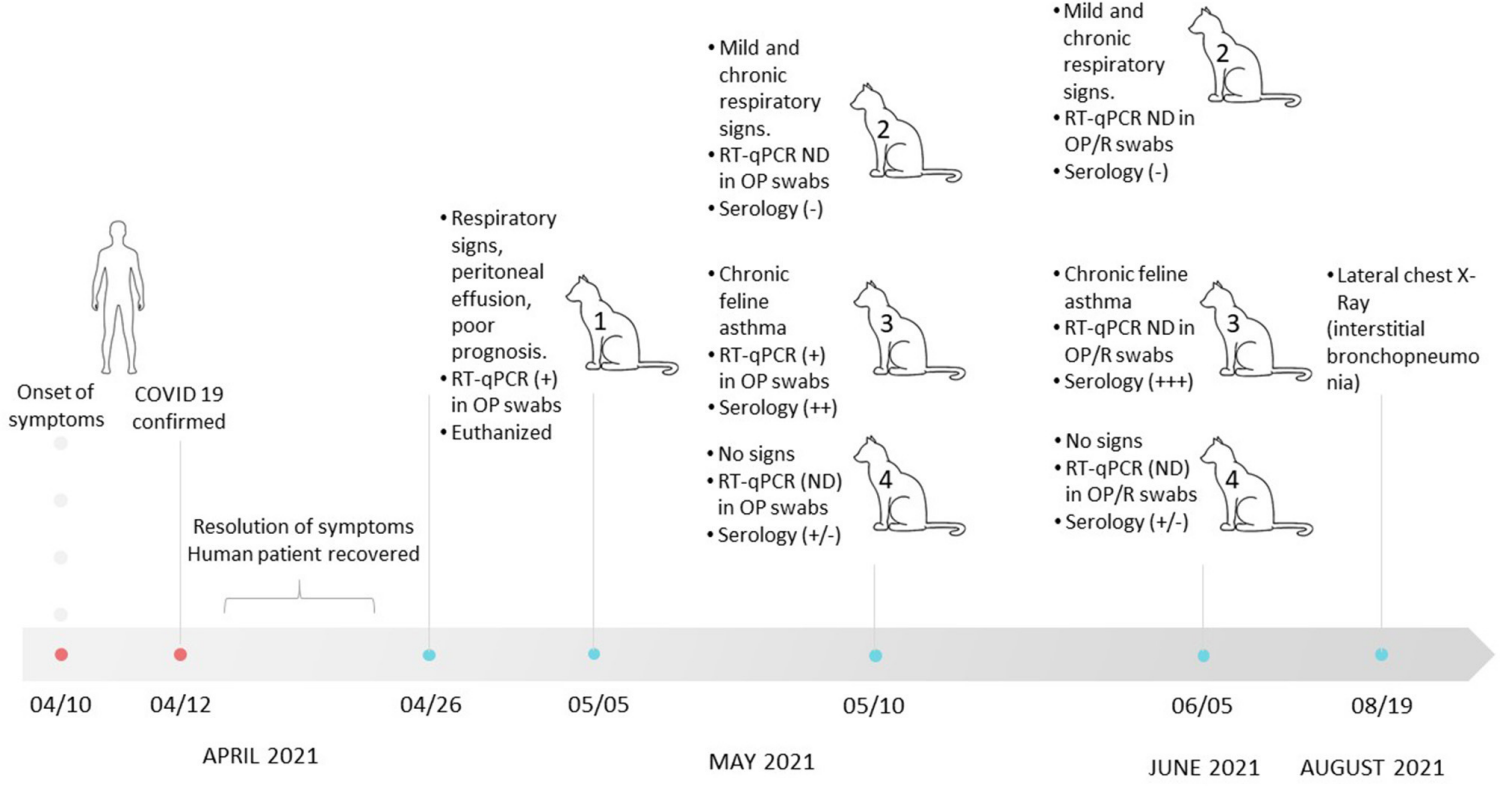
Timeline of clinical events involved in the SARS-CoV-2 infection of a human and household cats in Buenos Aires between March 10 and August 19. NP, nasopharyngeal; OP, oropharyngeal; R, rectal; ND, nondetectable.

On April 26, Cat N°1 presented with mild lethargy, and it was taken to the veterinary clinic. General examination showed 39.9°C of rectal temperature, and a mass was palpated in the mesogastrium region. In addition, an ultrasound exam showed peritoneal effusion and alteration of liver and kidney normal structures. SARS-CoV-2 infection was confirmed by RT-qPCR from the OP sample. Due to its bad health condition aggravated by peritonitis (with FIP-negative result by RT-qPCR (28), this cat was euthanized (Figure 1), and no further studies could be conducted.

The cat owners (family composed of three members) suffered from COVID-19 16 days before this episode with the cat. The owners developed clinical signs compatible with SARS-CoV-2 infection, and 2 days after the onset of symptoms, one of them tested detectable by RT-qPCR, with a VL of 5.8 × 10^7^ copies/μl. The other two cohabiting relatives were directly referred to as confirmed cases of COVID-19 without molecular testing.

Infection of Cat N°1 prompted the veterinarian to examine the other cats that shared the house. On May 10 and June 5, the three cats (N°2, N°3, and N°4) were evaluated. Cat N°4 presented with good general health in both clinical examinations. Cat N°2 was not febrile, with good body condition score, but showed a productive cough and chronic purulent rhinitis, which persisted until the date of writing of this manuscript. This clinical picture had started in the middle of 2020; therefore, it was not attributable to the coronavirus pandemic. In both cats, SARS-CoV-2 genome could not be detected at any time. Cat N°3 showed dry cough (feline asthma), which persisted until August 19, when a lateral chest radiograph was performed. The radiograph exam showed an increased bronchial and interstitial pattern marked in the diaphragmatic area compatible with interstitial bronchopneumonia (Supplementary Figure 1). This cat tested positive for the presence of SARS-CoV-2 by RT-qPCR on May 10. VLs were high in both cats (2.27 × 10^6^ copies/μl for Cat N°3 and 5.26 × 10^5^ copies/μl for Cat N°1; Figure 1 and Table 1).

**Table 1:** Summary of SARS-CoV-2 clinical presentation, RT-qPCR results, viral load, and partial Sanger sequencing in human and cat patients.

**Patient**	**Date collected**	**Sample type**	**Clinical signs**	**Days from the human onset of symptoms**	**RT-qPCR (Ct value)**	**Viral load (gen E copies/μl)**	**Variant (Spike gene sequencing)**
Human	4/12/2021	NP swab	Yes	N/A	15	5.8 10^7^	B1.1.7 (ALPHA)
Cat N 1	5/5/2021	OP/R swab	Mild lethargy, hyperthermia	25	20,8	5.26 10^5^	N/A
		peritoneal effusion			ND	N/A	N/A
Cat N°2	5/10/2021	OP swab	Productive cough and chronic purulent rhinitis	30	ND	N/A	N/A
	6/5/2021	OP swab		56	ND	N/A	N/A
Cat N°3	5/10/2021	OP swab	Dry cough	30	19	2.27 10^6^	B1.1.7 (ALPHA)
	6/5/2021	OP swab		56	ND	N/A	N/A
Cat N°4	5/10/2021	OP swab	No	30	ND	N/A	N/A
	6/5/2021	OP swab		56	ND	N/A	N/A

*NP, nasopharyngeal; OP, oropharyngeal; R, rectal; Ct, cycle threshold; ND, non-detectable; N/A, not applicable*.

### Serologic Results

For Cats N°2, N°3, and N°4, serum samples were collected 30 and 56 days after the onset of symptoms in the human patient and 14 and 40 days after Cat N°1 required veterinary assistance. Detection of antibody anti-spike protein by ELISA showed a strong positive response in Cat N°3 at both time points analyzed. Sera from the other two cats were not reactive in the indirect ELISA.

Pseudo neutralization assay (pNA) was carried out with serum samples collected on May 10. Both ELISA and pNA showed concordant results, and Cat N°3 was the only one showing detectable neutralizing antibodies against SARS-CoV-2 (Table 2). In addition, serum samples from Cats N°2, N°3, and N°4 resulted negative for feline coronavirus by immunochromatography data not shown.

**Table 2:** Detection of anti-spike antibodies by indirect ELISA and neutralizing antibodies by pseudo neutralization assay.

**Animal ID**	**ELISA**		**pNA**	
	**5/10/2021**	**6/5/2021**	**absIC80**	**absIC50**
CAT 2	0.051 ± 0.004	0.061 ± 0.001	ND	ND
CAT 3	2.478 ± 0.031	2.217 ± 0.077	1,461	797
CAT 4	0.181 ± 0.013	0.165 ± 0.002	ND	ND

*Anti-spike IgGs were assessed by indirect ELISA at two timepoints; results are expressed as OD measured at 450/630 nm. Neutralizing antibodies were evaluated only at the first time point, and the results are expressed as 50 and 80% inhibition (absIC50 or absIC80)*.

### Sequence Analysis

**Figure 2:**
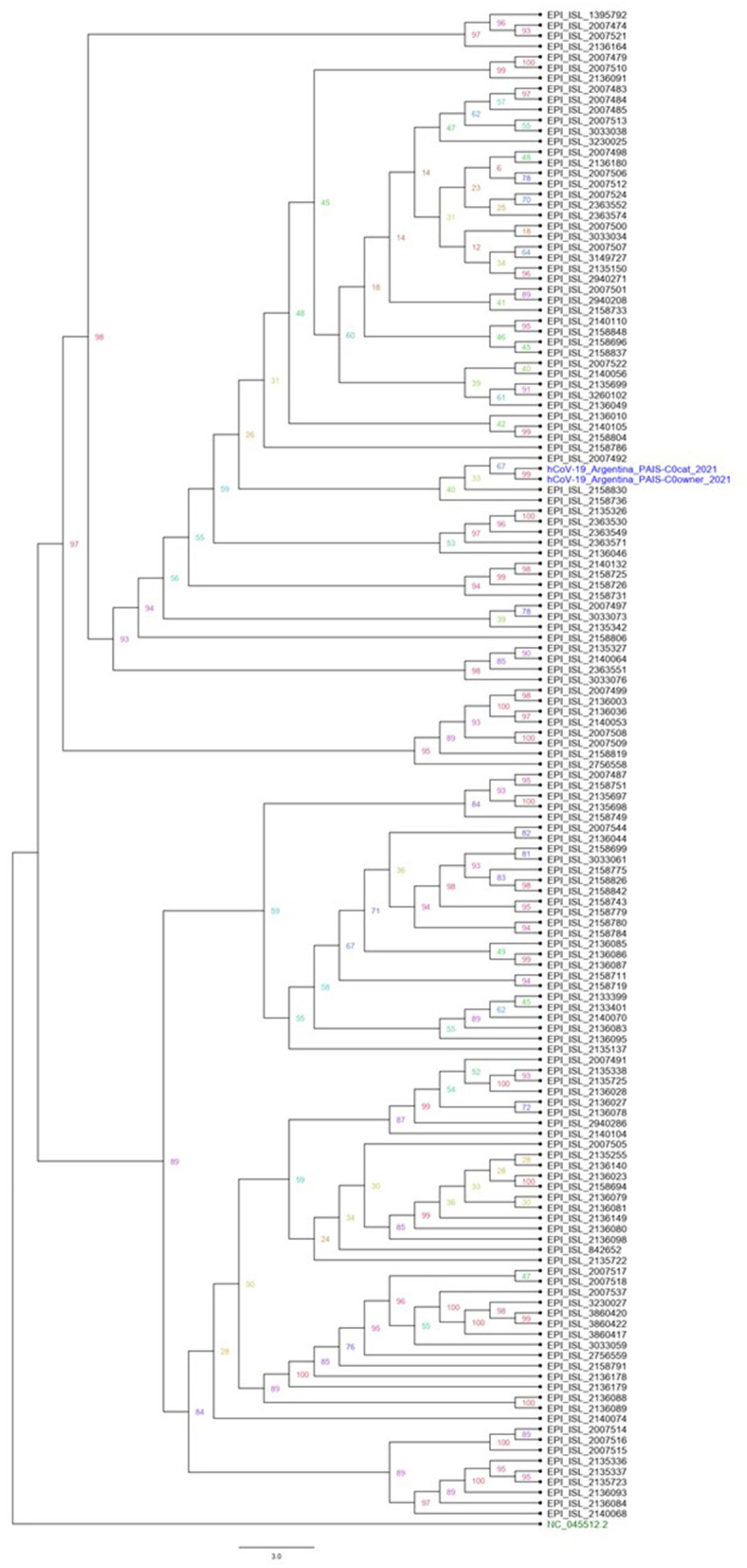
Phylogenetic analysis of Argentine SARS-CoV2 whole-genome sequence lineage B1.1.7. A maximum likelihood phylogenetic tree was performed using *IQ-TREE*. All Argentinian alpha sequences are available in the GISAID database. The datasets generated and analyzed in this study can be found in the GISAID repository (https://www.gisaid.org/). Accession numbers are EPI_ISL_4875630 and EPI_ISL_4875726.

The sequencing of the complete genome from human and cat samples indicated the presence of the alpha variants (lineage B1.1.7). In addition, an extra mutation, the C22997A (numbered according to the Wuhan sequence), was identified as unique to these two samples, which results in the amino acid change P479T in spike glycoprotein. The ML phylogenetic tree for Argentine SARS-CoV2 Alpha variant whole genome shows that hCoV-19/Argentina/PAIS-C0owner/2021 and hCoV-19/Argentina/PAIS-C0cat/2021 (human and cat samples, respectively) are closely related (Figure 2).

## Discussion

SARS-CoV-2 is efficiently transmitted from human to human, and there is consensus that this is the driving force of the current pandemic. Although animals are usually involved in zoonotic and emerging diseases, current knowledge suggests that wild and domestic animals do not play an active role in SARS-CoV-2 transmission to humans. Reverse zoonosis of SARS-CoV-2, however, is likely to occur since human infection is much more frequent than domestic animal infection. Moreover, infected owners that are in close proximity to their pets during acute infection can transmit the virus to them. In this report, we described a reverse zoonosis event that occurred between a human and two household cats in Buenos Aires, Argentina.

The pets that inhabited the house were adopted stray cats that were adults at the time of this study, and three of them had preexisting chronic respiratory signs as comorbidity. Cat N°1 was the first to show some symptoms compatible with COVID-19, and the presence of SARS-CoV-2 was confirmed a few days later. Cat N°2 and Cat N°3 showed mild clinical signs, and the last one gave a clear SARS-CoV-2-positive result by RT-qPCR. It is important to mention that 101 days after this result, a radiograph exam was conducted in Cat N°3 showing interstitial bronchopneumonia. A similar pattern was observed by Zoccola et al. (18) in a cat infected with SARS-CoV-2 alpha variant.

Taking into account that the four cats had been in close contact with each other, and the moment in which the virus was detected in the owner, in Cat N°1 and in Cat N°3, the possibility of transmission between cats in the household cannot be ruled out. Cat-to-cat transmission is feasible through direct contact or aerosols, which has been demonstrated in experimental infections (17, 21, 29–31).

Available research supports the low probability of pet-to-human or human-to-pet transmission (32); nevertheless, the potential role of companion animals in spreading SARS-CoV-2 to people in close contact with them or even as a reservoir for the virus should be clarified. This is particularly relevant since people infected with SARS-CoV-2 may remain asymptomatic during infection. Some reports indicate that this may be the same for animals (29–31); thus, it cannot be excluded the possibility that they could spread the virus without displaying any clinical sign of the disease. Moreover, if VLs in the pets were high, as in this study, the probability of virus transmission to humans or other animals could increase.

There is scarce data regarding the amount of virus present in infected pets, and until the writing of this work, to our knowledge, no one had reported the VL in positive samples. In most studies, the cycle threshold values determined in samples from infected animals were high. Although different methodologies are used in each work, the Cts associated with the detection of N, Orf1ab, and RdRP genes were higher than 30 (9, 11, 16, 18, 33).

Unlike previous works, in this study, VLs in both cats were as high as VL quantified in the human sample (5.26 × 10^5^ gc/μl for Cat N°1 and 2.27 10^6^ gc/μl for Cat N°3). Our results are in agreement with those reported by Hamer et al. (34), who detected, in one cat with mild clinical signs, Cts values ranging between 17.7 and 27.73 (corresponding to N, E, and RdRP genes). In this regard, the high VL in the human sample (5.8 × 10^7^ genomic copies/μl) could be responsible for viral transmission to the household cat (or cats).

The presence of SARS-CoV-2 in pets was also indirectly identified in several studies, by detecting specific antibodies against the spike protein or neutralizing antibodies (NA). In this work, sera from Cat N 3 presented a strong reactivity in an ELISA that detected anti-spike antibodies and showed a clear induction of NA. In accordance with our results, NA after SARS-CoV-2 infection was also described in studies conducted in cats and dogs in France (35), USA (Texas) (34), Italy (36), and Brazil (16), with varied % of animals having NA. It is important to mention that seropositivity among pets from COVID-19-positive owners was significantly greater compared with those with owners of unknown status (35, 36). On the other hand, some studies showed low or null detection of NA in cats and dogs suggesting that human-to-cat transmission might be relatively infrequent (14, 15, 32, 37, 38).

Recent findings indicate that dogs and cats are also susceptible to SARS-CoV-2 variants identified in human beings (39–41). In order to demonstrate owner–pet contagion, we performed whole genome sequencing for both samples. First, we determined that viral genomes from humans and cats belong to the B.1.1.7 (alpha) lineage and, when comparing complete genomes of alpha sequences from Argentina, we noticed a nucleotide change (C22997A) that causes the P479T amino acid change in the glycoprotein spike. This mutation was found only in those two sequences. We could not find information about the P479T mutation, but since it is close to T478K, one of Delta's marker mutations, it is interesting to follow working on the characterization of these samples.

As in our study, the first case of SARS-CoV-2 infection with the alpha variant (B.1.1.7) was reported in a domestic shorthair cat and a black lab-mix dog from the same household in Texas, United States (40); in both pets, clinical sign (sneeze) started several weeks after tested positive. In another case, two cats and a dog were infected by SARS-CoV-2 B.1.1.7 variant in the United Kingdom. The animals did not exhibit respiratory signs, but developed atypical clinical manifestations, including cardiac abnormalities secondary to myocarditis (39). Interestingly, the first Italian case of a household cat infected by an alpha variant was recently described. The cat showed overt respiratory signs 1 week after the clinical onset of COVID-19 disease of the owners, who had been also infected by the same variant. Another study, conducted in the Metropolitan Area of Buenos Aires, Argentina, with the aim of investigating the infection of SARS-CoV-2 in pets from owners previously confirmed as COVID-19 positive, detected one positive cat. Complete sequence and phylogenetic analysis demonstrated that the SARS-CoV-2 genome belonged to the B.1.499 lineage. This lineage had been actively circulating in the metropolitan area of Buenos Aires (9).

Although SARS-CoV-2 variants have been detected in pets, the effect of the described variants in animals, in terms of transmissibility, disease severity, and pathogenesis is still unknown. In this regard, further studies are required to assess the impact of the newly emerged SARS-CoV-2 variants in the epidemiology of SARS-CoV-2 in animals.

One of the main concerns in Public Health is the possibility of domestic animals acting as reservoirs of SARS-CoV-2. For that to happen, the virus would have to circulate efficiently in the susceptible animal population and further have to be reintroduced into the human population (42). However, existing data strongly suggest that the detection of SARS-CoV-2 in domestic animals is more likely due to the disease spillover from humans to animals (43).

The Centers for Disease Control and Prevention (CDC) recommends that people with suspected or confirmed COVID-19 isolate from their pets, just as they would from other members of their household, to reduce the potential of human-to-animal transmission (https://www.cdc.gov/coronavirus/2019-ncov/animals/interim-guidance-managing-people-in-home-care-and-isolation-who-have-pets.html).

Considering the impact of the ongoing COVID-19 pandemic and the well-known relevance of animals in the epidemiology of coronaviruses (animal spillover, cross-species jumping, and zoonotic concerns), the One Health concept becomes extremely important, now more than ever. Public Health and Veterinary Services must share information and work together integrating human, animal, and environmental perspectives to efficiently address health issues.

## Data Availability Statement

The datasets presented in this study can be found in online repositories. The names of the repository/repositories and accession number(s) can be found in the article/Supplementary Material.

## Ethics Statement

The animal study was reviewed and approved by CICUAL (Comité Institucional de Cuidado y Uso de Animales de Laboratorio) 2021/08 Facultad de Ciencias Veterinarias, Universidad de Buenos Aires. Written informed consent was obtained from the owners for the participation of their animals in this study.

## Author Contributions

APec, DM, MDu, APer, MC, APu, MG, and CC carried out the experiments, with the collaboration of SM and AG. MDu and MM wrote the manuscript with the support from APec and DM. OV and OR attended to the cats. MDi managed the information of the human component of the case. SM, JM, and AG helped supervise the project. MDu and JM procured funding and supervised the project. All authors contributed to the article and approved the submitted version.

## Funding

This research was funded by Fundacion Argeninta, Diagnotest SRL, and PIDAE Proyectos de Investigación y Desarrollo de Áreas Estratégicas con impacto social—UBA 2020 (Res CS 2021 346 E UBA REC). This study received funding from Diagnotest SRL. The funder was not involved in the study design, collection, analysis, interpretation of data, the writing of this article, or the decision to submit it for publication.

## Conflict of Interest

DM is employed by Diagnogen S.A. JM is employed by Diagnogen S.A. and Diagnotest SRL. The remaining authors declare that the research was conducted in the absence of any commercial or financial relationships that could be construed as a potential conflict of interest.

## Publisher's Note

All claims expressed in this article are solely those of the authors and do not necessarily represent those of their affiliated organizations, or those of the publisher, the editors and the reviewers. Any product that may be evaluated in this article, or claim that may be made by its manufacturer, is not guaranteed or endorsed by the publisher.
