# Supplementary material for: Anthropogenic Infection of Domestic Cats With SARS-CoV-2 Alpha Variant B.1.1.7 Lineage in Buenos Aires
Source: Front Vet Sci. 2022 Mar 1;9:790058. doi: 10.3389/fvets.2022.790058 (PMC8925007; doi:10.3389/fvets.2022.790058)
Supplement: Supplementary file 1 [file Data_Sheet_1.pdf]

Supplementary Material

1.1 Supplementary Figures

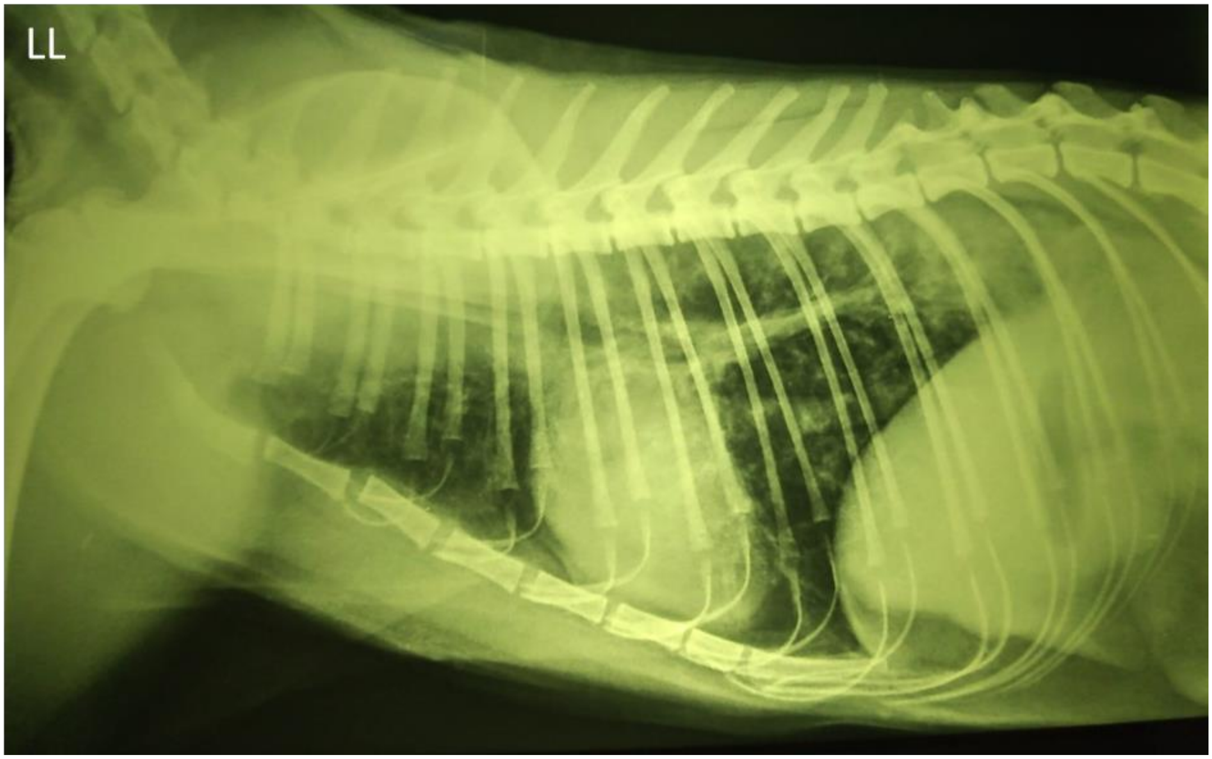

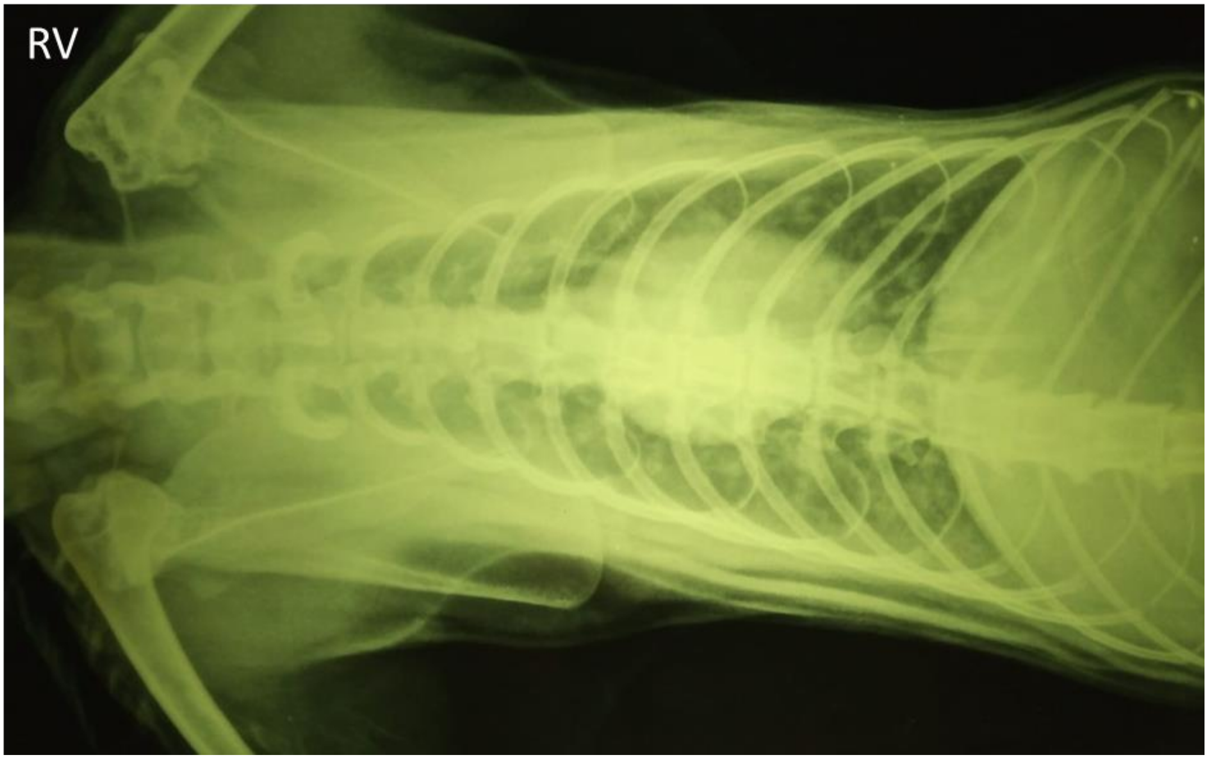

Supplementary Figure 1. Latero-Lateral (LL) and Ventrodorsal (RV) radiographic views of Cat Nº3.
